# Heightened cocaine-seeking in male rats associates with a distinct transcriptomic profile in the medial prefrontal cortex

**DOI:** 10.3389/fphar.2022.1022863

**Published:** 2022-12-14

**Authors:** Christina R. Merritt, Ashley E. Smith, Kamil Khanipov, George Golovko, Kelly T. Dineley, Noelle C. Anastasio, Kathryn A. Cunningham

**Affiliations:** ^1^ Center for Addiction Research, University of Texas Medical Branch, Galveston, TX, United States; ^2^ Department of Pharmacology and Toxicology, University of Texas Medical Branch, Galveston, TX, United States; ^3^ Department of Neurology, University of Texas Medical Branch, Galveston, TX, United States

**Keywords:** cocaine use disorder, cocaine-seeking, medial prefrontal cortex, RNA-sequencing, transcriptomics, 5-HT_2C_R

## Abstract

Drug overdose deaths involving cocaine have skyrocketed, an outcome attributable in part to the lack of FDA-approved medications for the treatment of cocaine use disorder (CUD), highlighting the need to identify new pharmacotherapeutic targets. Vulnerability to cocaine-associated environmental contexts and stimuli serves as a risk factor for relapse in CUD recovery, with individual differences evident in the motivational aspects of these cues. The medial prefrontal cortex (mPFC) provides top-down control of striatal circuitry to regulate the incentive-motivational properties of cocaine-associated stimuli. Clinical and preclinical studies have identified genetic variations that impact the degree of executive restraint over drug-motivated behaviors, and we designed the present study to employ next-generation sequencing to identify specific genes associated with heightened cue-evoked cocaine-seeking in the mPFC of male, outbred rats. Rats were trained to stably self-administer cocaine, and baseline cue-reinforced cocaine-seeking was established. Rats were phenotyped as either high cue (HC) or low cue (LC) responders based upon lever pressing for previously associated cocaine cues and allowed 10 days of abstinence in their home cages prior to mPFC collection for RNA-sequencing. The expression of 309 genes in the mPFC was significantly different in HC vs. LC rats. Functional gene enrichment analyses identified ten biological processes that were overrepresented in the mPFC of HC vs. LC rats. The present study identifies distinctions in mPFC mRNA transcripts that characterizes individual differences in relapse-like behavior and provides prioritized candidates for future pharmacotherapeutics aimed to help maintain abstinence in CUD. In particular the Htr2c gene, which encodes the serotonin 5-HT_2C_ receptor (5-HT_2C_R), is expressed to a lower extent in HC rats, relative to LC rats. These findings build on a plethora of previous studies that also point to the 5-HT_2C_R as an attractive target for the treatment of CUD.

## Introduction

Recent sharp rises in cocaine-related overdoses (Ahmad et al., 2022) associate with a history of cocaine use disorder (CUD) in some individuals (Yoon et al., 2014; John and Wu, 2017; Yule et al., 2018). Recidivism rates are high even among CUD individuals who have undergone rehabilitation (Brandon et al., 2007), and the field currently lacks approved pharmacotherapy to aid in sustaining recovery. Abstinence is challenged by sensitivity to stress, withdrawal symptoms, and cocaine-associated stimuli (e.g., cocaine-associated cues) which can trigger craving and relapse (Hendershot et al., 2011). Functional top-down control of the medial prefrontal cortex (mPFC) over striatal circuitry titrates the incentive-motivational properties of cocaine-associated stimuli (McLaughlin and See, 2003) and mPFC neuroadaptations resulting from chronic cocaine use associate with increased relapse vulnerability (for reviews) (Kalivas, 2005; Van den Oever et al., 2010; Jasinska et al., 2014; Cunningham et al., 2020). Preclinical analyses have illustrated persistent changes in mPFC gene transcripts resulting from cocaine exposure and withdrawal (Freeman et al., 2008; Walker et al., 2018). However, variation within the mPFC transcriptome contributory to individual differences in vulnerability to cocaine-seeking remains underexplored. Genes associated with heightened cue-evoked cocaine-seeking could serve as prioritized targets for neuropharmacological intervention to support abstinence and mitigate relapse in CUD patients particularly vulnerable to cocaine cue-evoked relapse.

The present study quantified cocaine-seeking following a 10-days period of abstinence from cocaine self-administration in outbred, male Sprague-Dawley rats to detect “low cue” (LC) and “high cue” (HC) phenotypes. Rats were categorized into phenotypes based on the degree of lever-presses for cocaine-associated cues. Next-generation sequencing (NGS) and bioinformatics analyses were utilized to identify neurobiological signatures within the mPFC transcriptome that correlate with heightened sensitivity to cocaine-associated cues (Geschwind and Konopka, 2009; Wang et al., 2009; Volkow et al., 2015). We established a unique mPFC transcriptomic landscape in the of HC vs. LC rats which validates known, and identifies, new molecular mechanisms to be interrogated in CUD relapse vulnerability.

## Materials and methods

### Animals

Male, Sprague-Dawley rats (*n* = 12; Envigo, Houston, TX) weighing 250–275 g upon arrival were housed two per cage and maintained on a 12-h light-dark cycle with monitored and controlled temperature (21°C–23°C) and humidity (45%–50%). Rats were acclimated to the colony room for 7 days prior to handling and experimentation and were allowed *ad libitum* access to food and water except during daily operant sessions. Rats were 11 weeks old at the start of operant training. Experiments were carried out in accordance with the National Institutes of Health Guide for the Care and Use of Laboratory Animals (2011) and with the approval of the University of Texas Medical Branch Institutional Animal Care and Use Committee.

### Cocaine self-administration

Rats (*n* = 12) were anesthetized (8.6 mg/kg of xylazine, 1.5 mg/kg of acepromazine, 43 mg/kg of ketamine in bacteriostatic saline) prior to surgical implantation of indwelling jugular catheters with back mounts; rats were allotted 7 days to permit postoperative recovery (Cunningham et al., 2011; Cunningham et al., 2013; Anastasio et al., 2014a; Anastasio et al., 2014b). Rats received a 0.1 ml infusion of a bacteriostatic saline solution containing heparin sodium (10 µ/ml; American Pharmaceutical Partners, East Schaumburg, IL), streptokinase (0.67 mg/ml; Sigma Chemical, St. Louis, MO), and ticarcillin disodium (66.67 mg/ml; Research Products International, Mt. Prospect, IL) into the catheter immediately following daily self-administration sessions to ensure catheter patency during experimentation.

The cocaine self-administration assay utilized standard operant conditioning chambers (Med Associates, Inc., St. Albans, VT) housed within sound-attenuated, ventilated cubicles equipped with fans (Med Associates, Inc.). Operant chambers were fitted with two retractable response levers, a stimulus light above each response lever, a houselight on the wall opposite the response levers, and an external pellet dispenser. In 180-min sessions, rats were trained to perform a lever press response reinforced by infusions (0.75 mg/kg/0./1 ml) of (-)-cocaine HCl (NIDA Drug Supply Program, Bethesda, MD) dissolved in 0.9% NaCl and delivered through syringes loaded daily into infusion pumps (Med Associates, Inc.) (Cunningham et al., 2011; Anastasio et al., 2014a; Anastasio et al., 2014b; Swinford-Jackson et al., 2016). The infusion pumps were connected to liquid swivels (Instech, Plymouth Meeting, PA) fastened to catheters *via* polyethylene 20 tubing encased inside a metal spring leash (Plastics One, Roanoke, VA).

Scheduled responses on the active lever resulted in the delivery of a cocaine infusion over a 6-s period; each infusion was simultaneously paired with the illumination of the house and stimulus lights and activation of the infusion pump (i.e., discrete cue complex paired with cocaine delivery). Inactive lever presses were recorded but had no scheduled consequences. The stimulus light and infusion pump were inactivated following the delivery of cocaine. The house light remained on to signal a timeout period (20 s); lever presses committed during the timeout period had no scheduled consequences. Rats were trained on a fixed ratio (FR) 1 schedule of reinforcement and progressed to a FR5 schedule until stability as assessed by achieving <10% variation in the number of infusions and lever presses for a minimum of three consecutive sessions (within-subjects (phenotype), repeated measures (day), two-way ANOVA; GraphPad Prism 9.2.0) (Cunningham et al., 2013; Anastasio et al., 2014a; Anastasio et al., 2014b; Sholler et al., 2019; Anastasio et al., 2020).

### Cue-reinforced cocaine-seeking

**FIGURE 1:**
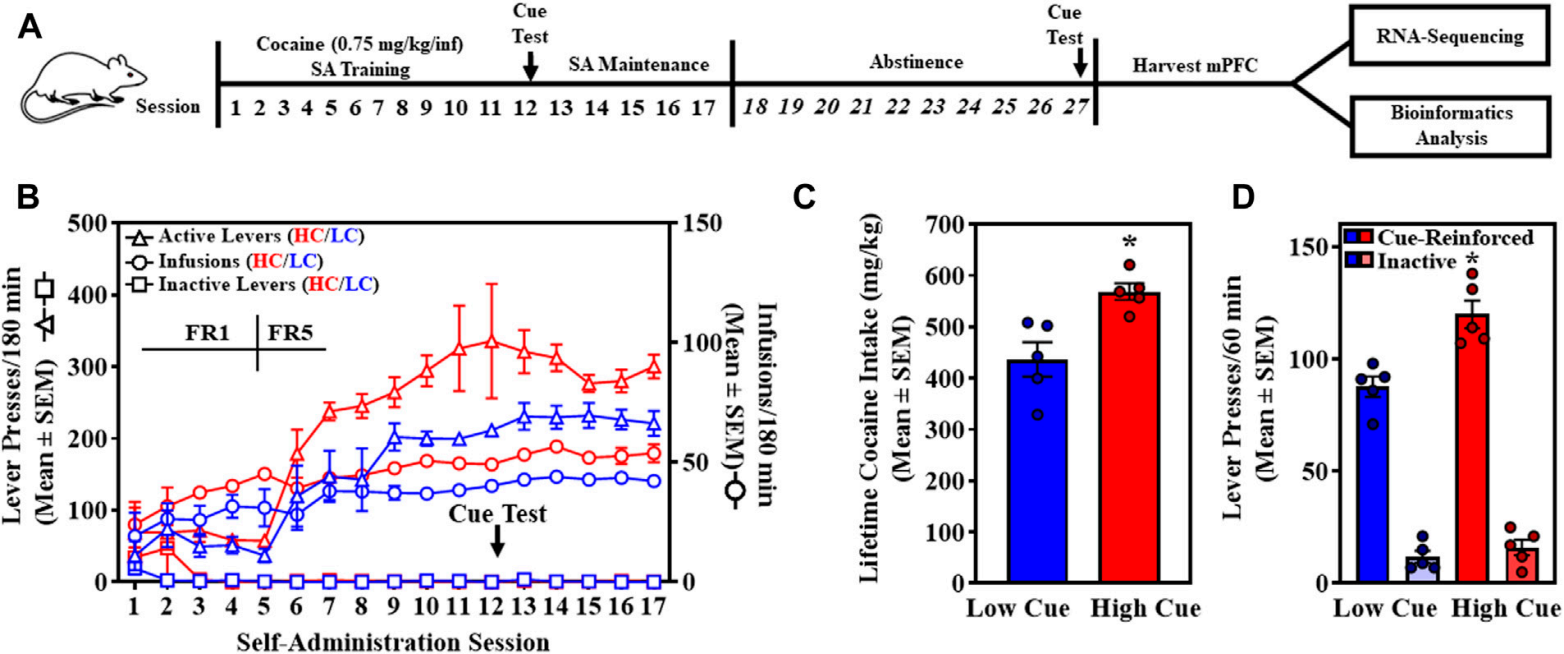
High cue (HC, red) and low cue (LC, blue) rats are stratified by levels of cue-reinforced lever presses in the self-administration (SA) model prior to transcriptomic analyses. **(A)** The experimental timeline is shown. **(B)** The time course of self-administration sessions is presented as the total presses (mean ± SEM) on the active (black circles; left *Y*-axis) or inactive lever (white circles; left *Y*-axis), and total number of cocaine infusions obtained in the 180-min session (purple circles; right *Y*-axis). **(C)** Total lifetime cocaine intake (mg/kg; mean ± SEM) is presented for HC (red) and LC (blue) rats. An unpaired, one-tailed Student’s *t*-test revealed HC rats (*n* = 5) self-administered a significantly higher amount of cocaine (568.31 ± 56.12 mg/kg) relative to LC rats (436.34 ± 33.49 mg/kg) (t_8_ = 3.546, *p* = 0.0076) (unpaired, two-tailed, Student’s *t*-test). **(D)** Total cue-reinforced lever and inactive lever responding during the first cue test on Day 12 (mean ± SEM) are presented for HC (red) and LC (blue). An unpaired, one-tailed Student’s *t*-test revealed HC rats (*n* = 5) displayed significantly higher lever presses (119.8 ± 6.21) for cocaine-associated cues relative to LC rats (*n* = 5; 87.6 ± 4.57) (t_8_ = 4.178, *p = 0.0015). An unpaired, one-tailed Student’s *t*-test revealed inactive lever presses did not differ between HC (16.0 ± 3.49) and LC rats (*n* = 5; 11.8 ± 2.73) (t_8_ = 0.9477, *p* = 0.1855).

Once stable cocaine self-administration was achieved, individual levels of cue-reinforced cocaine-seeking were assessed in a 60-min session (see experimental timeline, Figure 1A). Lever presses on the previously active lever were reinforced by the cocaine-associated discrete cue complex (stimulus light illuminated, infusion pump activated) in the absence of cocaine, on a FR1 schedule. Inactive lever presses were recorded with no scheduled consequences. Rats were stratified as HC or LC rats based on a median split analysis of the total number of cue-reinforced lever presses on session 12 (unpaired, one-tailed Student’s *t*-test) (Anastasio et al., 2014a; Anastasio et al., 2014b; Fink et al., 2015; Sholler et al., 2020).

Self-administration resumed following the cue probe and continued daily for an additional five maintenance sessions, followed by 10 days of abstinence in home cages and daily handling (Figure 1A). Half of the rats from each phenotype (*n* = 3/phenotype) were randomly selected and a second cue-reinforced cocaine-seeking test was conducted to interrogate maintenance of HC and LC phenotypes on session 27 (Pearson’s correlation). Total lifetime cocaine consumption [daily intake (mg/kg) = infusions per session * cocaine infusion dose (mg/ml) * infusion volume (0.1 ml/infusion) * body weight (kg)-1] for HC and LC rats was also determined (unpaired, two-tailed Student’s *t*-test).

### RNA isolation from mPFC tissue homogenates

Rats (*n* = 12) were anesthetized with chloral hydrate (400 mg/kg) on experimental day 27, and brains were dissected. The mPFC, comprised of infralimbic, prelimbic, and anterior cingulate cortex regions (from Bregma: 3.2–1.2 mm anteroposterior, ±1.2 mm mediolateral, −5.2 mm dorsoventral) (Paxinos and Watson, 2005), was microdissected immediately over ice, flash-frozen in liquid nitrogen, and stored at −80°C for subsequent RNA isolation as previously described (Anastasio et al., 2014a; Anastasio et al., 2014b; Fink et al., 2015; Sholler et al., 2020). The mPFC was homogenized in 10 X W/V extraction buffer (20 mM HEPES, 200 mM NaCl, 1 mM EDTA, 1 mM DTT, 10 μl/ml protease inhibitor cocktail, 10 μl/ml phosphatase inhibitor cocktails 2 and 3 (Sigma-Aldrich, St Louis, MO), and 5 μl/ml RNaseOUT™ Recombinant Ribonuclease Inhibitor (Thermo Fisher Scientific, Waltham, MA). Immediately following homogenization, the sample was transferred to 500 μl of TRI Reagent (Life Technologies, Grand Island, NY), and isolated RNA was purified using RNeasy Mini Kit (Qiagen, Germantown, MD). The RNA concentration and quality were analyzed using Cytation 5 Cell Imaging Multi-Mode Reader (BioTek Instruments, Winooski, VT) and preserved at −80°C until assayed.

### RNA-sequencing and bioinformatics pipeline

Total RNA-sequencing was performed at the University of California Los Angeles Technology Center for Genomics and Bioinformatics. Libraries were prepared using Kapa Stranded RNA-Seq Kit (Kapa Biosystems, Wilmington, MA). During the library preparation step, samples underwent enrichment, cDNA conversion, end repair, A-tailing, multiplexing, and amplification. Next, libraries were sequenced as single-end, 50 base-pair reads using the Illumina Hiseq3000 instrument generating between 16 and 39 million reads per sample. Two samples (*n* = 1/phenotype) did not meet quality control standards and were excluded from all analyses, and our final sample size was ten (*n* = 5/phenotype). The literature supports a minimum of five biological replicates per experimental group for the conduct of transcriptomics analyses *in vitro* and *ex vivo* (Ching et al., 2014). CLC Genomics Workbench v21 (Qiagen Bioinformatics) (Sholler et al., 2020) was used for sequencing quality control and mapping of the RNA-Seq data to the *Rattus norvegicus* reference genome and the collection of the coding sequences (Sholler et al., 2020). Sequencing data were initially trimmed using CLC’s “Trim Reads” module. Reads containing nucleotides below the quality threshold of 0.05 (using the modified Richard Mott algorithm), those with two or more unknown nucleotides or sequencing adapters were trimmed from the dataset. Filtered sequencing reads were then processed using the “RNA-Seq Analysis” module by mapping using a global alignment strategy against the Rat Rnot_6.0.97 annotated reference genome with 95% length fraction and 80% similarity fraction scores. Differential gene expression analysis was performed as part of the “RNA-Seq Analysis” module. The gene expression count was modeled by a separate Generalized Linear Model (GLM), and statistical significance was evaluated using the Wald Test. A volcano plot of differentially expressed genes was rendered using the Volcano Plot tool in Galaxy Version 0.0.5 (Afgan et al., 2018). Functional enrichment analysis was performed separately using Ingenuity Pathway Analysis (IPA) (QIAGEN, Venlo, NL) to determine the biological functions of significantly differentially expressed genes. The most significant functional pathways [−log10 (*p*-value) ≥ 1.3] were then evaluated. The genes associated with behavioral pathways by IPA were extracted and further evaluated using the Cytoscape (Shannon et al., 2003) application ClueGo (Bindea et al., 2009). As a result, we remapped the significant, differentially expressed genes to a more common open source using Gene Ontology accession numbers (Ashburner et al., 2000; Gene Ontology, 2021) and further dissected into more specific and relevant biological processes specifically related to behavior. The Search Tool for the Retrieval of Interacting Genes/Proteins (STRING) database (Version 11.5; ELIXER, Cambridgeshire, United Kingdom) (Szklarczyk et al., 2019; Szklarczyk et al., 2021) was utilized to determine protein–protein interactions which are predicted based on published experimental data (e.g., co-expression, protein homology) and other bioinformatics databases [(e.g., Kyoto Encyclopedia of Genes and Genomes (KEGG)], Reactome, etc.) to generate the protein-protein interaction (PPI) network [reference genome: *Rattus norvegicus*, confidence score = 0.4 (default).

## Results

### High cue and low cue rats are distinguished based upon cue-reinforced lever presses

Outbred rats trained to self-administer cocaine (0.75 mg/kg/infusion) acquired stable self-administration by experimental session 11 (Figure 1B). Across the last three sessions, a two-way ANOVA (factors: phenotype, between-subjects and day, within-subjects) revealed a main effect of phenotype on infusions earned (F_1,8_ = 38.01, *p* = 0.0003), but no main effect of day (F_2,16_ = 0.9057, *p* = 0.4240) or significant phenotype X day interaction (F_2,16_ = 0.8262, *p* = 0.4555). These results indicate differences in cocaine-taking between phenotype, but not across the days preceding the cue test indicating stable self-administration. Total cocaine intake (Figure 1C; mean ± SEM) was higher in HC rats (568.31 ± 56.12 mg/kg) relative to LC rats (436.34 ± 33.49 mg/kg) (t_8_ = 3.546, *p* = 0.0076) (unpaired, two-tailed, Student’s *t*-test).

Rats were stratified as exhibiting greater than (HC rats) or less than (LC rats) the median (102.5) of total cue-reinforced lever presses on the first cue assessment on session 12, respectively (*n* = 5/phenotype) (Figure 1D) (Anastasio et al., 2014a; Anastasio et al., 2014b; Fink et al., 2015; Sholler et al., 2020). An unpaired, one-tailed Student’s *t*-test revealed total cue-reinforced lever presses (mean ± SEM) were significantly higher in the HC (119.8 ± 6.21) relative to the LC (87.6 ± 4.57) phenotype (t_8_ = 4.178, *p* = 0.0015). Total inactive lever presses (mean ± SEM) were not different between HC (16.0 ± 3.49) and LC (11.8 ± 2.73) rats (t_8_ = 0.9477, *p* = 0.1855) (unpaired, two-tailed Student’s *t*-test). On experimental day 27, cue-reinforced lever presses were reassessed in a subset of randomly selected rats (*n* = 6) and a positive correlation was observed between their two assessments (*R* ^2^ = 0.75, *p* = 0.013), indicating stability of the HC and LC phenotype (Pearson’s correlation analysis).

### RNA-sequencing reveals 309 genes in the mPFC differ between HC and LC rats

**FIGURE 2:**
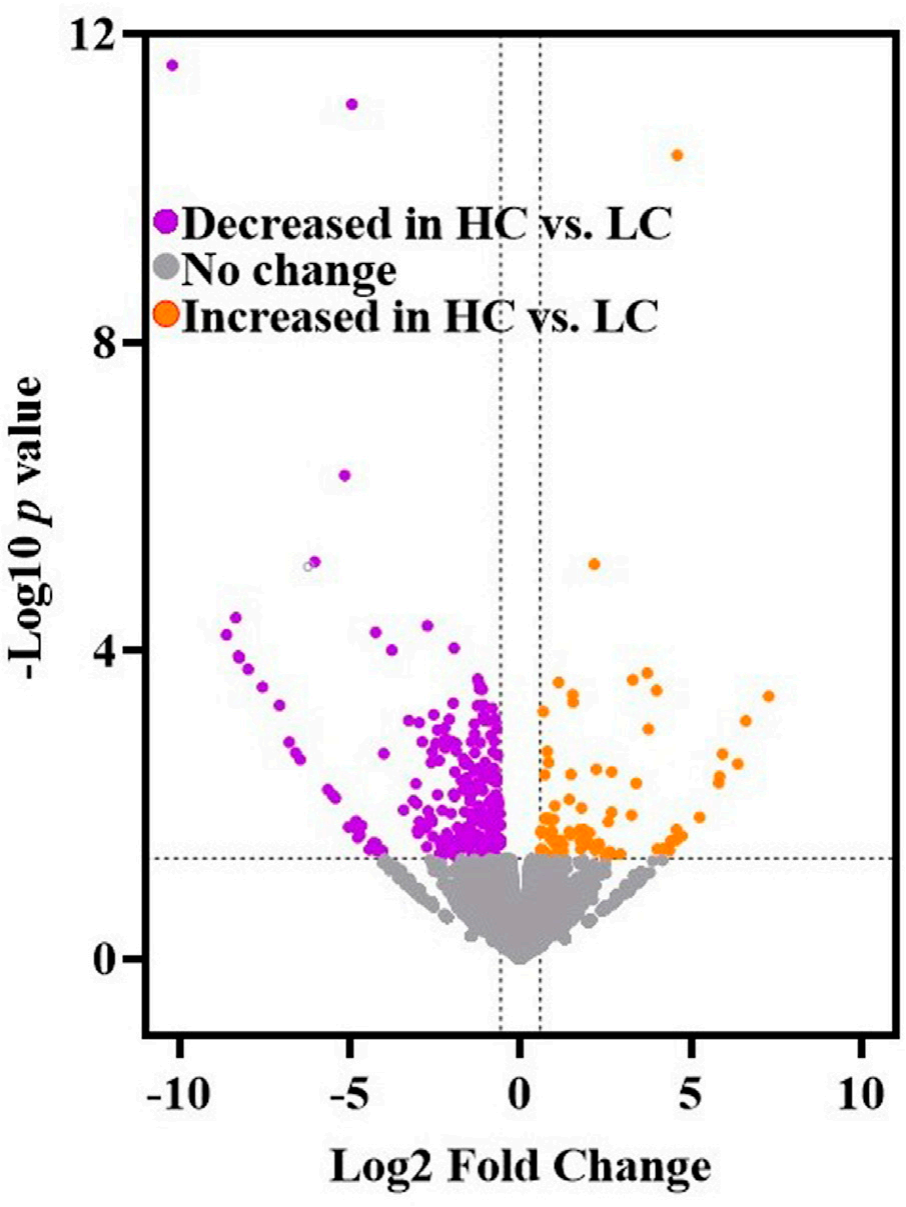
The volcano plot displays the results of mPFC RNA-seq results (CLC Genomics Workbench v21, Qiagen Bioinformatics) (Sholler et al., 2020) for HC and LC rats (generated by the Volcano Plot tool in Galaxy Version 0.0.5) (Afgan et al., 2018). Purple symbols (*n* = 227) indicate genes with lower expression in the mPFC of HC vs. LC rats. Orange symbols (*n* = 82) indicate genes with higher expression in mPFC of HC vs. LC rats. Significance criteria include -Log10 *p*-value ≥1.3 (*y*-axis) and Log2 fold change ≤ −0.58 or ≥ +0.58 (*x*-axis) (see Methods).

Genes that were significantly increased or decreased in the mPFC of HC rat relative to LC rats was determined by analyzing gene expression count with a Generalized Linear Model (GLM) followed by the Wald Test (CLC Genomics Workbench v21, Qiagen Bioinformatics) (Sholler et al., 2020). A volcano plot of differentially expressed genes was rendered using the Volcano Plot tool in Galaxy Version 0.0.5 (Afgan et al., 2018). The expression of 309 genes in the mPFC significantly differed between HC vs. LC rats [-Log10 *p*-value ≥1.3 (*y*-axis) and |Log2 Fold Change| ≥ 0.58 (*x*-axis)]. The volcano plot (Figure 2) displays genes with lower (purple symbols; *n* = 227) and higher (orange symbols; *n* = 82) expression in the mPFC of HC vs. LC rats.

### Gene enrichment identifies biological processes overrepresented in the mPFC of HC rats

**FIGURE 3:**
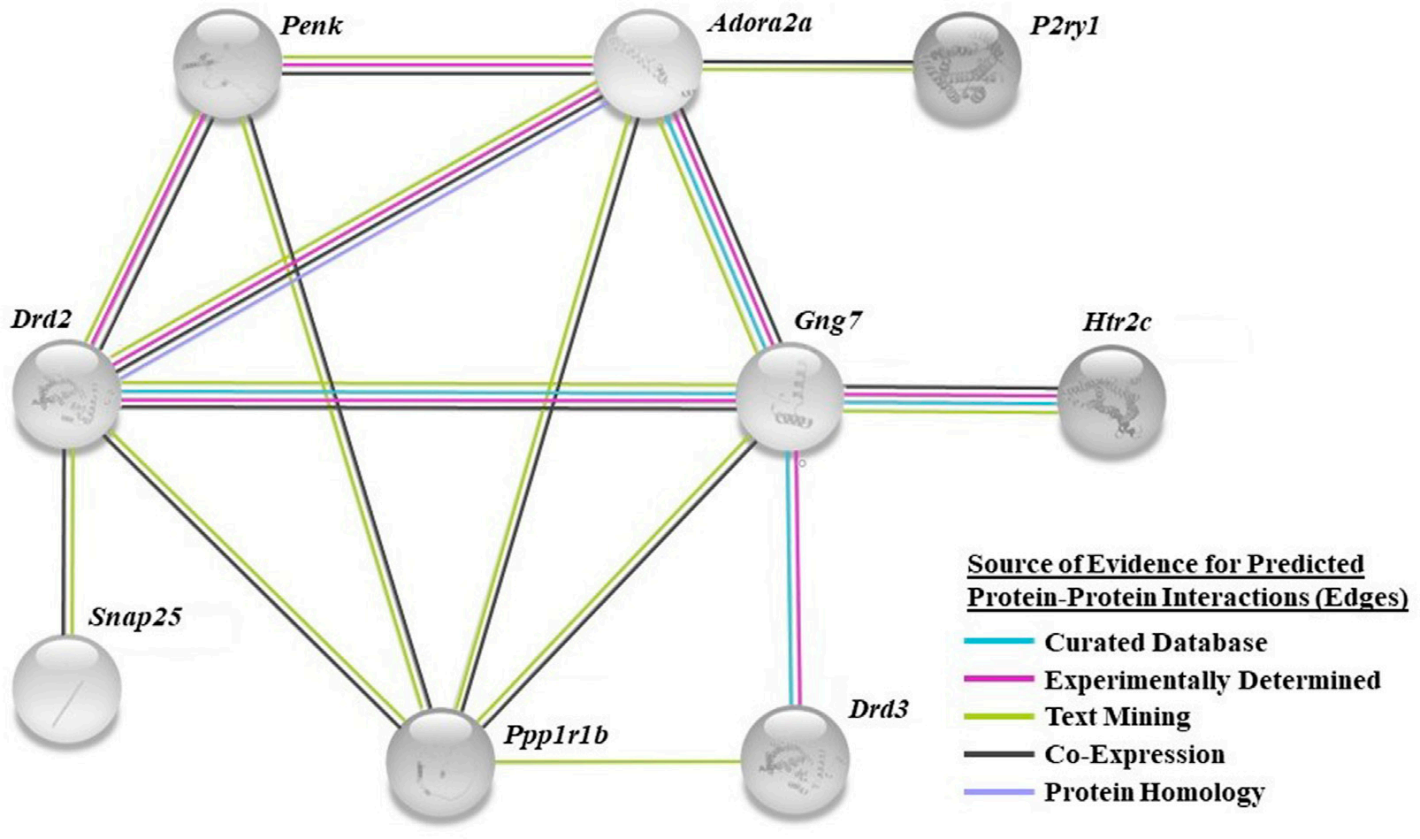
STRING bioinformatics analysis identified a network of predicted interactions in a subset of the selected genes. The specific color of network edges corresponds to the source of evidence for each known or predicted interaction (source: teal, curated database; magenta, experimentally determined; green, text mining; black, co-expression; lavender, protein homology). Confidence threshold based upon an interaction score of 0.4 minimum (see Methods).

We utilized a combined gene set enrichment analysis approach with Ingenuity Pathway Analysis (**IPA**) (QIAGEN, Venlo, NL) and ClueGO (Cytoscape plug-in) to provide biological context to the registry of 309 differentially expressed genes (Bindea et al., 2009). A cluster of ten biological processes associated with behavior was found to be overrepresented in the HC phenotype (*p* < 0.05) (Table 1, top left column). Eighteen differentially expressed genes were found to populate these biological processes (Table 1). For example, adenosine receptor subtype 2A (*Adora2a*), cell cycle exit and neuronal differentiation 1 (*Cend1*), dopamine D2 receptor (*Drd2*), dopamine D3 receptor (*Drd3*), fibroblast growth factor 14 (*Fgf14*), serotonin 5-HT_2C_ receptor (*Htr2c*), proenkephalin (*Penk*), protein phosphatase 1 regulatory inhibitor subunit 1B (*Ppp1r1b*), and synaptosome associated protein 25 (*Snap25*), and thyroid stimulating hormone receptor (*Tshr*) associate with locomotor behavior (GO:0007626). The computed false discovery rate (FDR)-adjusted *p*-values for behavior-related genes suggest a possibility of type I error, however the literature supports the involvement of these genes in CUD (see Discussion). A STRING network analysis (Version 11.5; ELIXER, Cambridgeshire, United Kingdom) (Szklarczyk et al., 2019; Szklarczyk et al., 2021) reveals predicted PPIs between nine of the 18 highlighted targets (e.g., *Adora2a-Drd2*, Figure 3). Network nodes (proteins) (Aci-SeChe et al., 2014) are shown with one or more connections (edges) to representing each line of evidence that predict an interaction between two nodes (e.g., protein homology; see Figure legend).

**TABLE 1:** Functional gene enrichment (IPA plus Cytoscape ClueGO) reveals behavior-related biological processes (top, left) that are overrepresented in the mPFC of HC vs. LC rats, significance and associated genes noted in upper middle and right columns, respectively. Full gene names for highlighted genes are noted below. Fold change values (bottom, right) for associated genes (bottom, left) that populate these biological process pathways are presented with significance noted (bottom, middle). Significance criteria include fold change >1.0, *p*-value <0.05 (see methods).

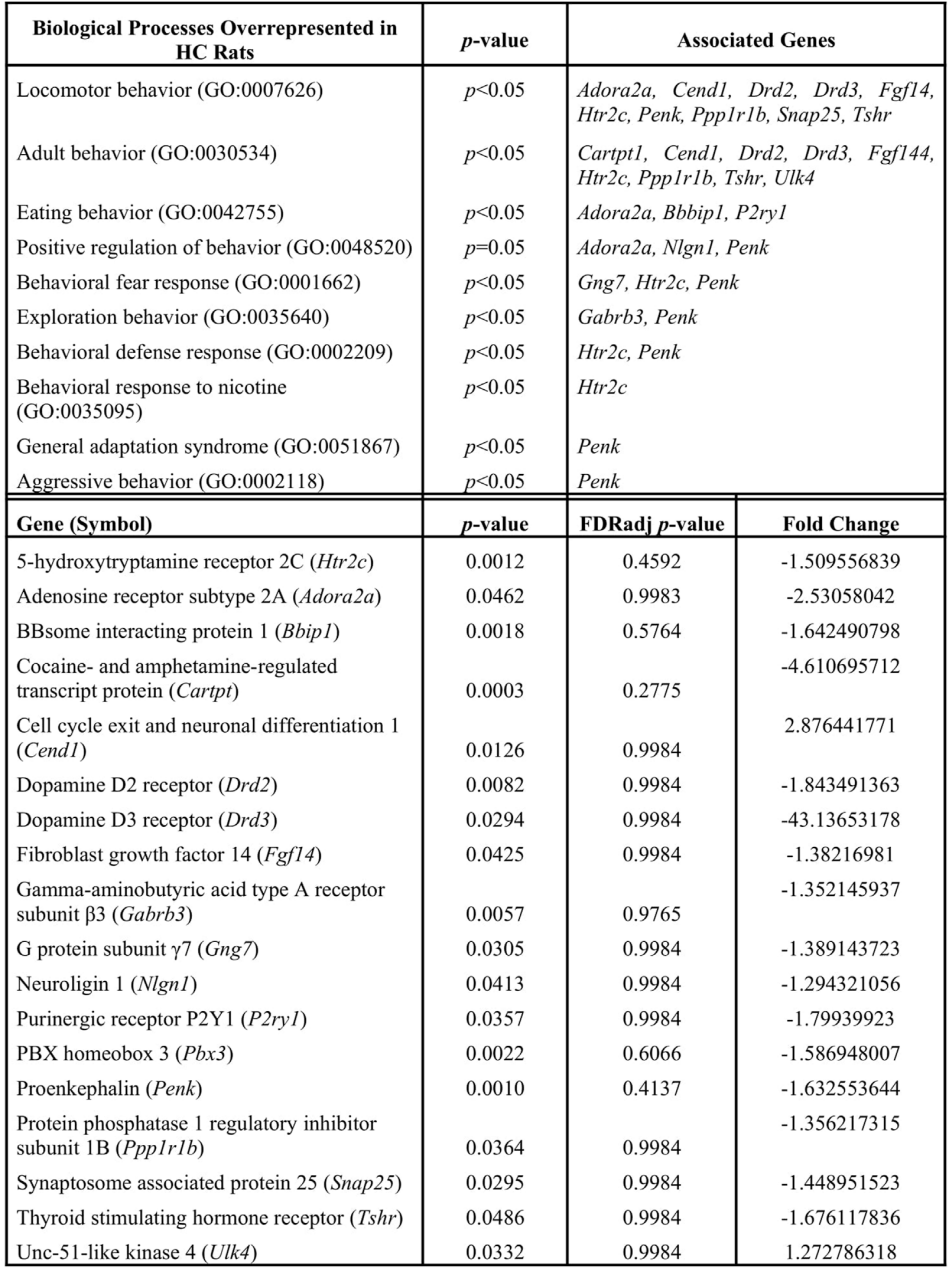

## Discussion

We provide an unbiased evaluation of gene regulation in the mPFC associated with individual differences in cocaine-seeking during abstinence in male, outbred rats. Our interrogation isolated several candidate genes associated with heightened cue-evoked cocaine-seeking. Intriguingly, out of the 309 differentially expressed genes, the majority (∼73%) were decreased in the mPFC of HC rats. It is plausible this translated to a loss of fine-tuned, top-down regulation of downstream striatal circuitry implicated in drug-seeking behavior. Gene enrichment analyses narrowed our focus to genes that contribute to behavior-related biological processes (*n* = 18). We identified overarching themes (e.g., hyperactivity, heightened stress) connecting several biological processes overrepresented in the HC phenotype. These global themes might have been expected as hyperactivity and heightened stress states are often comorbid in individuals diagnosed with CUD, especially in the preoccupation/anticipation stage modeled here (Koob and Kreek, 2007). Here, we specifically highlight genes implicated in several biological processes (e.g., *Htr2c*) or predicted to interact at the protein level (e.g., *Adora2a*, *Drd2*). Our laboratory (Anastasio et al., 2013; Felsing et al., 2018; Price et al., 2019; Soto et al., 2019) and others (Tao et al., 2017; Hámor et al., 2018; Hasbi et al., 2020) have identified biologically meaningful PPIs which promise to enrich our understanding of SUD mechanisms and perhaps serve as targets for study in the therapeutic space.

The *Htr2c* gene encodes the serotonin (5-HT) G-protein coupled receptor (GPCR) 5-HT_2C_ receptor (5-HT_2C_R) and our transcriptomic analyses identified lower *Htr2c* mRNA expression in the mPFC of abstinent HC vs. LC rats (FC: 1.510), aligning with lower 5-HT_2C_R protein expression previously observed in the mPFC during abstinence (Anastasio et al., 2014b; Swinford-Jackson et al., 2016). An investigational 5-HT_2C_R agonist evinced efficacy, but reduced potency, to inhibit cocaine-seeking during abstinence (Swinford-Jackson et al., 2016), supporting the concept that 5-HT_2C_R agonists (Fletcher et al., 2004; Cunningham et al., 2011; Harvey-Lewis et al., 2016; Anastasio et al., 2020), may ameliorate hypofunctional 5-HT_2C_R neurotransmission as a core mediator of relapse vulnerability (for reviews) (Howell and Cunningham, 2015; Cunningham et al., 2020). However, while the 5-HT_2C_R agonist lorcaserin reduced craving in non-treatment seeking CUD participants (Pirtle et al., 2019; Johns et al., 2021), this medication may facilitate continued abstinence in treatment-seeking CUD participants (NCT03007394). While these outcomes may reflect translational challenges in medication development for CUD, future studies are required to define specific molecular strategies to enhance performance of cortical 5-HT_2C_R function to mitigate relapse in CUD patients. For example, a 5-HT_2C_R positive allosteric modulator is sufficient to suppress cocaine-seeking in male rats (Wild et al., 2019), perhaps due to its superior selectivity for the 5-HT_2C_R over the other 5-HT_2_ receptors (Wold et al., 2019) relative to lorcaserin (Thomsen et al., 2008).

Eight Bardet-Biedl Syndrome (BBS) proteins form the BBsome complex, a stable complex involved in signaling receptor trafficking to and from ciliary membranes of neurons (Nachury et al., 2007; Scheidecker et al., 2014). The *Bbip1* gene encodes one of these proteins (BBSome interacting protein 1) (Loktev et al., 2008) which facilitates vesicular trafficking of GPCRs from cytosolic compartments to the plasma membrane in neurons (Domire et al., 2011; Guo et al., 2016; Shinde et al., 2020). Our transcriptomic analysis identified the *Bbip1* gene as expressed in the mPFC to a lower degree in HC rats vs. LC rats (FC: 1.642). Interestingly, genetic disruption of the BBsome complex stalled 5-HT_2C_R trafficking in late endosomal compartments, resulting in decreased 5-HT_2C_R ciliary membrane expression in neurons (Guo et al., 2019). This induced a biologically significant impact such that the anorectic effect of the 5-HT_2C_R agonist lorcaserin was blunted in a rodent model of obesity (Guo et al., 2019). It is possible that reduced *Bbip1* expression led to diminished BBsome-mediated receptor trafficking and further disruption of 5-HT_2C_R function in HC rats. In line with this hypothesis, we demonstrated that the ratio of membrane to cytoplasmic 5-HT_2C_R protein expression in the mPFC is inversely correlated with cocaine-seeking (Swinford-Jackson et al., 2016). Future studies are required to determine the exact impact of *Bbip1* loss and BBsome complex disruption on 5-HT_2C_R trafficking in key neuronal circuitry involved in cocaine-motivated behaviors.

A core network of predicted PPIs generated by the STRING biological database shows several layers of association (edges) between nine of the eighteen genes of interest. Genes coding for the adenosine A2A receptor (*Adora2a*; FC: 2.531), dopamine receptor D2 (*Drd2*; FC: 1.843), G protein subunit gamma 7 (*Gng7*; FC: 1.389), and protein phosphatase 1 regulatory inhibitor subunit 1B (*Ppp1r1b*; FC: 1.356) were the most interconnected nodes each with edges reaching to five other genes in the network. In contrast to the current findings, a separate study found *Adora2a*, *Drd2*, and *Ppp1r1b* to be increased in the mPFC of “vulnerable” mice that exhibit heightened cocaine-motivated behavior, relative to “resilient” mice; phenotypes were assigned to each animal through multi-factor analysis of operant behavior observed during cocaine self-administration sessions (e.g., cocaine intake) (Navandar et al., 2021). While both studies implicate these genes to be important regulators of cocaine-motivated behaviors, differential gene expression patterns could be expected to be dependent on the given stage of CUD being modeled (active drug consumption vs. abstinence). The adenosine 2A (A2A) and dopamine D2 (D2) receptors, predicted to physically interact in the current PPI analysis, form heterodimers within the striatum (Prasad et al., 2021; Valle-Leon et al., 2021). Activation of the A2A receptor blocks D2 receptor-mediated inhibition of striatal neurons when in complex, while A2A receptor antagonists enhance dopaminergic signaling (Azdad et al., 2009). The A2A-D2 receptor heteromer is under investigation as a therapeutic target for disorders associated with hypodopaminergic tone (i.e., CUD) (Parsons et al., 1991; Moeller et al., 2012). Most of this research is focused within the striatum; the functional impact of this association within cortical neurons remains underexplored.

Our experimental design allowed for the identification of 309 genes differentially expressed in the mPFC within male rats displaying differential responsiveness to cues previously associated cocaine intake. Intriguingly, but perhaps not surprisingly, we observed that total lifetime cocaine intake associated with the highest level of cue-evoked cocaine-seeking. This is consistent with previous observations that demonstrate greater levels of cocaine exposure associate with heightened CUD-like behavior (e.g., cocaine-primed cocaine-seeking) in rodents (Deroche-Gamonet et al., 2004). Conceivably, it is plausible that the transcriptomic differences detected are directly linked with overall heightened cocaine-motivated behavior during both self-administration (cocaine-taking) and cue test (cocaine-seeking) sessions. A recent publication sought to identify the transcriptomic changes occurring in several mesocorticolimbic nodes in rats trained to self-administer saline or cocaine, animals were euthanized 24 h or 30 days following the final self-administration session (Walker et al., 2018). Although animals in the current study were euthanized at 10 days of abstinence from cocaine, the genes we highlight here still do not overlap with those identified as differentially expressed in the PFC in saline *versus* cocaine self-administration rats. This suggests variation in cocaine-seeking, but not cocaine exposure, is the primary driver of transcriptomic differences in mPFC of HC rats. Although, the discrepancy between these two findings may be related to species and/or timepoint differences. As we have demonstrated in the past, mechanisms associated with self-administration of cocaine are distinct relative to non-drug rewards such as sucrose (Swinford-Jackson et al., 2016). Inclusion of a sucrose self-administration control group in the future would be useful in parceling out neuromolecular drivers of drug and natural reinforcers. Lastly, female rodents frequently exhibit heightened psychostimulant-seeking following cessation of self-administration relative to male rats (for review) (Becker and Koob, 2016). However, the impact of chronic cocaine exposure and variation in cocaine-seeking on the mPFC transcriptome of female rats is underexplored; future studies are warranted to determine if our findings are generalizable across sex. In conclusion, we propose the 5-HT_2C_R, BBsome complex, and A2A-D2 receptor heteromer should be further investigated as prioritized candidates for the development of pharmacotherapeutics to reduce relapse vulnerability.

## Data Availability

The datasets presented in this study can be found in online repositories. The names of the repository/repositories and accession number(s) can be found below: https://www.ncbi.nlm.nih.gov/geo/, GEO accession: GSE196374.

## Abbreviations

5-HT, serotonin; 5-HT_2C_R, serotonin 5-HT_2C_ receptor; ANOVA, analysis of variance; BBS, Bardet-Biedl Syndrome; CUD, cocaine use disorder; FR, fixed ratio; GEO, Gene Expression Omnibus; GLM, Generalized Linear Model; GPCR, G protein-coupled receptor; HC, high cue; IPA, Ingenuity Pathway Analysis; KEGG, Kyoto Encyclopedia of Genes and Genomes; LC, low cue; mPFC, medial prefrontal cortex; NGS, next-generation sequencing; PPI, protein-protein interaction; RPKM, reads per kilobase per million; SA, self-administration; STRING, Search Tool for the Retrieval of Interacting Genes/Proteins.
